# Supplementary material for: Attitudes, perceptions, and preferences towards SARS CoV-2 testing and vaccination among African American and Hispanic public housing residents, New York City: 2020–2021
Source: PLoS One. 2023 Jan 19;18(1):e0280460. doi: 10.1371/journal.pone.0280460 (PMC9851504; doi:10.1371/journal.pone.0280460)
Supplement: S1 File — (DOCX) [file pone.0280460.s001.docx]

**Focus group guide**

**Introduction**

Hi everyone. Thank you for coming today. My name is ____ [name]. I am a ____ [title] from New York University. As you may know, there is a large effort underway to increase testing for coronavirus across NYC. We are conducting focus groups with NYCHA residents to gain a better understanding of your thoughts about COVID-19 testing, including your experiences, your concerns about testing, your preferences for where and when to be tested and any challenges you have experienced or others you know have experienced in trying to get tested. I want to start by acknowledging that everyone here has most likely had a range of different experiences over the last 6 months. Some of you may have been sick. Others may have had family and close friends become ill or have someone close die of COVID-19. We are interested in hearing about these experiences, including how they shape your thoughts about testing. But we also want to stress that participating in this group discussion does not mean that you have to answer questions or talk about anything that makes you uncomfortable.

The information you share today will remain anonymous. So please do not use anyone’s names during our group discussion. We ask that you “raise your hand” on Zoom if you want to speak.

Your participation is totally voluntary which means that you can decide to leave the Zoom meeting at any time. You may not benefit from the research personally, but we hope that the information that you share will improve our understanding of how to expand uptake of COVID-19 testing to reduce spread of disease.

Our group discussion will take about 60 minutes. We will record our conversation so we can accurately capture all information that you will share with us. The recording will be transcribed and then erased and the transcription will be saved on a password-protected computer that is only accessible to the research team. When we present the findings from this group discussion they will be only presented as a summary so the information cannot be linked to any one person who is participating.

**Ground rules**

Before we start, I’d like to set up some rules to make sure all of our voices are heard and respectful.

- Please turn your phones in the mute mode. If you have to make a call, you can leave and do it out of the “room” and on mute.
- Let’s make sure that we speak one at a time and be respectful of everyone’s opinions. There are no right or wrong answers.
- We want to hear from all of you. Please make sure that you give others a chance to speak.

Does anyone have any questions? Shall we start?

*****************************************************************************************************************

**A. General pandemic experience**

*I’d like to start by talking a little bit about the pandemic in general.*

A.1. Can you describe your experiences since the pandemic started? ***PROBE****: how affected, illness, job disruptions, fears, financial impact*

A.2. Can you share some examples of how NYCHA has responded to COVID-19? ***PROBE*:** *what efforts NYCHA staff/management made to support residents? Disseminated masks, informational sessions, food distribution*

A.3. What do you think about these response efforts? ***PROBE****: what do you think about the services, usefulness of information?*

A.4. What about residents? How have residents in your development responded to the pandemic? ***PROBE****: specific examples of what residents have done independent of the development leadership*

*Thank you for sharing this information. I’d like to turn now to talking specifically about testing for COVID-19.*

**B. Knowledge about testing**

B.1. Can you share what you know about COVID in general? ***PROBE:*** *for example, where did it come from? How is it spread? Are residents calling it COVID or is there another term?*

B.2. Where are you getting most of your information about COVID? ***PROBE****: most common sources, which sources do you trust most?*

B.3. Let’s talk about testing. Can you share what you know about COVID-19 testing? ***PROBE****: for example, how is the test done, where you can get tested, why get tested, safety of testing, accuracy of testing*

B.4. Where would you go if you wanted to get tested?

**C. Attitudes toward testing**

C.1. What do you think about getting tested? ***PROBE****: what are your concerns, privacy concerns, fears of getting a positive test? What do you think are the advantages of getting tested? What are the disadvantages?*

C.2. Can you share some reasons why you or someone you know has gotten tested?

C.3. For those of you who have not been tested, what are some of the reasons you haven’t been tested? ***PROBE****: concerns about job loss, what are reasons you are hearing generally why people are not getting tested, e.g., costs, insurance status*

C.4. If you haven’t been tested, or haven’t been tested in a while, what would make you decide to get tested in the near future? ***PROBE****: symptoms, family members ill, work requirements, doctor recommendation/referral*

**D. Impact of positive results**

D.1. How would getting a positive test affect you and your family? ***PROBE:*** *lost wages, lost job, fear of infecting my family/coworkers*

D.2. What would be the challenges to isolating yourself if you were positive? ***PROBE***: *How might you deal with those challenges? What would you need?*

**E. Strategies for increasing testing**

E.1. What could the city or NYCHA do to address residents’ concerns about testing? *PROBE: more information from trusted source, resources if we have to stop working, a place to go to protect my family*.

E.2. The city has made several services available for people who need to isolate themselves. These include hotels that are completely paid for and food delivery and other support while you are isolated. Had anyone heard about this? Would this make it more likely that you would get tested knowing that if you were positive there is a place to stay?

E.3. What else do you think we can do to expand testing among residents? ***PROBE:*** *types of support services like CHWs that would encourage testing*

E.4. What are the best ways to raise awareness about testing options? ***PROBE***: *NYCHA-hosted events and activities to raise awareness, social media, TV ads*, *what messages*

E.5. What can residents do to increase awareness?

**F. Testing experience**

F.1. What challenges have you experienced in getting a COVID test or trying to get tested? ***PROBE****: Don’t know where to get tested, location of testing not convenient, having someone to take you to the location, fear of getting positive results*

F.2. For those of you have been tested, can you describe the experience? ***PROBE***: *positive and negative, long waits? Concern about exposure while waiting, why did they decide to get tested?* ***NOTE****: ask about when they were last tested and where they were tested*

F.3. What has worked well in terms of the testing experience and process? ***PROBE***: *what made it easier to get tested, what made the experience positive, what made it negative? How could the experience be improved?*

F.4. For those of you who have been tested, what was it like to get the test results? ***PROBE:*** *how did you get them? Were the results clear to you? Did you feel they were sensitive to confidentially? What was the timeframe in getting results and was it as expected?*

**G. Preferences for testing**

*I’d like to talk now about your preferences related to the testing process. (NOTE: some of this may have been answered in previous questions)*

G.1*.* Where would you prefer to get tested? ***PROBE****: home, mobile van, clinic, tents onsite,* ***PROBE****: explore what they think are advantages and disadvantages to each.*

*G.2. Do you have a preference for who would do your test?* ***PROBE****: training, race/ethnicity, doctor, nurse, lay health worker*

G.3. The current tests are done in a couple of ways. For example, they can be done by taking a swab from inside the nose, also called a nasal swab, and using saliva (describe each briefly). What would you prefer? Why? ***PROBE***: *not as accurate, challenges of returning the test*

**H. Contact tracing**

H.1. In addition to expanding testing, the city is conducting a contact tracing program. How much do you know about this? *(NOTE: let them respond and then expand on definition)*

H.2. What do you think about this program? ***PROBE***: *concerns, positives, negatives*

H.3. If you were to test positive, how willing would you be to share your contacts’ names and information? Why, why not, and what would encourage you to share contact information?

**I. Resident ideas about reducing and eliminating COVID**

I.1. I wanted to end by asking you, What do you think needs to happen to significantly reduce the risk or even eliminate COVID-19 from your development and community?

I.2. We understand there may be mistrust and fear related to COVID testing. Can you tell us where that comes from? ***PROBE:*** *news, government, medical doctors, members of the community, social media*

I.3. What do you suggest as good ways to reach residents who don’t trust the health care system and/or are fearful of testing?

**J. Vaccine**

*Many of you may know that there are many companies developing a vaccine. The results from the vaccine trials suggests that at least one vaccine will be approved and become available early next year.*

J.1. What do you think about vaccinations in general? ***PROBE:*** *why get vaccinated? What are the risks? Probe if they get annual flu shot and why or why not*

J.2. How do you feel about getting a COVID-19 vaccine? ***PROBE:*** *concerns, challenges*

J.3. Who would you trust to learn about the safety of a new vaccine or how effective it was?

**Wrap up**

I’d like to end by asking if you have any other comments.

That is all of my questions. Thank you for sharing your thoughts and time.
