# Supplementary material for: Attitudes, perceptions, and preferences towards SARS CoV-2 testing and vaccination among African American and Hispanic public housing residents, New York City: 2020–2021
Source: PLoS One. 2023 Jan 19;18(1):e0280460. doi: 10.1371/journal.pone.0280460 (PMC9851504; doi:10.1371/journal.pone.0280460)
Supplement: Attachment — Submitted filename: RADx FG PLoS One_Response to Reviewers_final.docx [file pone.0280460.s002.docx]

October 15, 2022

Dear Dr. Liu,

Thank you for reviewing our manuscript and for the opportunity to revise it to meet PLoS One’s publication standard. Please find listed below the comments submitted by the reviewers and our response to each comment in bolded italicized font. Please let us know if we can clarify anything further.

**Reviewer #1**: Thank you for the opportunity to review an interesting paper with significant issues identified for increasing uptake of testing and vaccination of African American and Hispanic people. I do think that the manuscript would benefit from some changes and further considerations, which I outline for each section.

Introduction
The Introduction described the study well and what was done, as well as some brief consideration of the issues faced regarding testing with African American and Hispanic people. What is needed is some more consideration of the literature regarding the topics of testing and vaccinations - the Introduction is currently very focused on the project, rather than what literature informed the project. Some discussion of what we know from literature about the determinants/predictors of willing to be tested (and vaccinated) is needed. There may not be work with these specific populations, but there should be some work that has been done more generally.

***RESPONSE.*** *We updated the introduction to address the reviewer’s comments which included reviewing additional literature and moving sections that described the methods to that section. We agree with this reviewer’s comment that much of what was previously included in the Introduction is more fitting for the Methods section.*

**REVIEWER.** The Introduction should also end with a purpose statement. Rather than focusing on what was done (the focus groups), the purpose of the study - e.g., to examine African American and Hispanic people's perceptions of COVID testing and vaccinations, and barriers/enablers to both - would situate the paper more as a research-oriented paper.

***REPONSE.*** *We thank the reviewer for this comment and have added a purpose statement at the end of the Introduction section.*

**REVIEWER.** The Method was described well. More information is needed regarding how participants were recruited - for example, how were they recruited and how did they get in touch with the researchers.

***RESPONSE.*** *We have added more information including eligibility criteria and more detail on recruitment strategies.*

**REVIEWER**. Some example interview questions should be included in the Method.

***RESPONSE.*** *The interview questions will be provided in the supplemental appendix.*

**REVIEWER.** The analysis was said to use rapid qualitative methods. Some more information on the use of this approach and a justification is needed. I wondered why an approach such as thematic analysis was not utilized. It is also stated that a deductive approach was used - however, without theory or models and previous literature presented in the Introduction, it is difficult to know how this was done. The integrated behavior model is mentioned in passing in the Method, but if this was the underlying approach, this needs to be discussed in the Introduction.

***RESPONSE.*** *We have expanded the description of the theoretical framework that informed the focus group guide.* *Rapid qualitative methods and analysis is a well described approach that emerged from the need, in situations such as in the case of formative work, where the timeline is short and the data is needed to inform modifications to be used in a larger randomized controlled trial. We used the integrated behavioral theory framework to inform the questions that were asked. Then using those questions, we created a template for the deductive analysis.* *We used a team based, highly iterative process that did allow for additional codes to emerge. This process doesn’t preclude more inductive assessments in the future*

**REVIEWER.** Results. The results provide interesting data. I did think some more nuance in discussion of results was needed - at times, statements are made that make it sound like every participant agreed. Consider some more discussion of to what extent certain attitudes were prevalent. Some themes received very little attention - the theme 'Why residents are getting tested' was very short. At other times, statements are made very briefly - e.g. concerns about getting positive tests are given one sentence only.

***RESPONSE.*** *In many of the focus groups, there was agreement among the participants as it pertains to specific topics/themes, particularly among the African American participants. Where there were differences, we did highlight those themes, specifically in the last two paragraphs of the results section. For example, the results indicate that the Spanish language focus group participants did not report vaccine hesitancy and had much higher trust in mainstream information sources.*

**REVIEWER.** Focus group numbers and participant numbers should be included so the reader can see the diversity in use of participant responses.

***RESPONSE.*** *Focus group numbers have been added to participant responses. As part of the transcription process, any distinct or identifying information between participants is removed to ensure the data is completely de-identified. As such, we cannot provide individual participant numbers.*

**REVIEWER.** It is mentioned towards the end of the Results that there was a difference between African American and Hispanic responses. This perhaps should be mentioned earlier, if there was such a marked difference.

***RESPONSE.*** *We tried to recruit equal numbers of Hispanic and African American participants; however, Hispanic individuals were reluctant to enroll. This may have been due to the sociopolitical context of the time in terms of concerns about immigration status. As a result, we only had one Hispanic focus group. Due to the limited amount of data from this population, we highlight some of the clear difference, however, we cannot make any additional conclusions about substantive differences between the two groups****.***

**REVIEWER.** I did wonder if both attitudes to testing and attitudes to vaccines could be discussed together in each theme - e.g. the misinformation theme could include reflections regarding both - but I will leave this up to the authors to decide if they want to do this.

***RESPONSE.*** *RADx-UP was and is an initiative to increase SARS-Cov-2 testing. However, once we launched the study, the vaccine was made available so we included information about the vaccinations because participants were interested in discussing it. We agree that it wasn’t explored as robustly as the testing questioning, however, vaccination uptake/hesitation was included in this manuscript because it became a major point of discussion during the focus group sessions given the timing of the study which coincided with the emergency use approval of the first vaccinations against Sars-Cov-2 in the U.S.*

**REVIEWER.** Again, I would have liked to see, given the deductive approach, how it reflected parts of the models used. The models do not need to be explicitly mentioned in the Results, but there should be some connection between their description in the Introduction and then the themes in the Results.

***RESPONSE.*** *As per the response above, we have expanded on this section.*

**REVIEWER.** Discussion was well done. I would like to see more reference to COVID literature - again, this may not necessarily be from studies with African Americans and Hispanic people (if there are only a few), but issues such as attitudes to vaccines, questioning existence of the virus etc., have been investigated and the current results should be examined in light of these findings.

***RESPONSE.*** *This is addressed in the updated introduction.*

**REVIEWER.** Data availability. I wasn't sure if the transcripts need to be potentially available to other researchers (e.g. on request). I will let the Editor decide that as I am very familiar with the data policies of the journal.

***RESPONSE.*** *The data is available for review upon request****.***

**Reviewer #2**:  Minor revisions are suggested.
Introduction 69-70 highlights broad applications of the findings of this study. Were they also directly used to improve testing services by RADx, NYU, or other institutions or initiatives noted in the introduction?

***RESPONSE.*** *Yes, the study was completed to inform the development of an intervention to increase uptake of Sars-CoV-2 testing among African American and Hispanic residents in public housing.*

**REVIEWER**. Introduction. Provide context on where the nation was in terms of testing and vaccination availability at the time that this intervention was carried out. Were opinions shared on a real or hypothetical vaccine candidate? A bit on this is discussed in results but it should be discussed here.

***RESPONSE.*** *We have provided this information in the Methods section under the “Context” sub-heading. The focus groups began roughly a year after the start of the pandemic, by which point COVID tests were widely available and routine testing encouraged. The bulk of the discussions around vaccine happened after vaccines had been rolled out and approved for emergency usage by the FDA.*

**REVIEWER**

Introduction 59-67. This description of the focus group discussion design is likely more appropriate for the methods section. It would be helpful to include an explanation (including references) in the introduction on the importance of working with CBOs such as those in the design of such an intervention.
***RESPONSE***. *We thank the reviewer for this comment and we have moved this portion to the Methods section.*

**REVIEWER**
Introduction 63 "Collectively, they designed and conducted..." recommend rewording to clarify if "they" includes the authors/NYU because as it stands this seems to suggest this is referring to the CBOs.
***RESPONSE.*** *This is addressed in the updated Introduction.*

**REVIEWER.**  Methods 87-90. Please provide examples of focus group questions.

***RESPONSE***. *Focus group interview guide questions available in supplemental appendix*

**REVIEWER.** Methods. 92-94. Clarify what the authors "protocol-driven, deductive" approach was. What protocol or theory was informing the analysis? The referenced article does not add clarity to the statement. Were the domain names identified based on a particular guiding framework? Based on consultation with the CSC?

***RESPONSE.*** *See the previous response to this question. Given the nature of the COVID-19 pandemic and the need to generate actionable next steps to inform the final design of the intervention, the analysis was conducted using a rapid qualitative analysis process. The questions were drawn from integrated behavior model with guidance from the CSC, and the main code names were derived from the questions.*

**REVIEWER**. Results. Demographics. Was any participant demographic information beyond race collected that might help contextualize the study findings? Age, for example? In results 194-203 you seem to suggest most of the 8 focus groups were with African-Americans and 1 was with Hispanic Americans. It would be helpful to clarify the number of participants identifying as African-American and/or Hispanic American included in the sample.

***RESULTS***. *No other demographic information beyond race/ethnicity were collected under suggestion by a CBO partner because of concern that too many personal questions may be considered as too intrusive by the study population. This is a limitation of the study. As for the breakdown of the demographics, of our 36 participants 4 identify as Hispanic/Latino; 30 Black or African American, and 2 Asian.*

**REVIEWER.** Results. Suggest "development" -> "housing development"

***RESPONSE.*** *This is addressed in the updated manuscript****.***

**REVIEWER.** Discussion. 210-214. You describe differences with respect to some of these conclusions among Af-AM and His-Am participants in the results section- it will be important to preserve these distinctions in this section so as to not conflate the perceptions and experiences of these two groups inappropriately.

***RESPONSE.*** *We have highlighted the thematic distinction with supporting quotes between these two groups in the final two paragraphs of the results section.*

**REVIEWER.** Discussion 249-262. Some of this is new data that should be in the results, likely in the section titled "Strategies for increasing testing," not introduced for the first time in the discussion. Eg. "with the vaccine now widely available, residents who are vaccinated were confused about the indications for future testing."

***RESPONSE.*** *We have edited this in the discussion section.*

**Reviewer #3:**  The topic is important and interesting, but the manuscript is lacking in its framing in the introduction, the description of methods, and valuable interpretation/contextualization in the discussion. It also has significant readability issues. Specific comments for each section follow: Introduction:- The authors should describe and cite existing literature on perceptions towards testing and vaccination among marginalized groups
***RESPONSE.*** *We have added additional references to the literature****.***

**REVIEWER**- The last sentence of the introduction is the first time that vaccination is mentioned - why did the study also include perceptions towards vaccination? The authors need to provide framing and justification for this

***RESPONSE.*** *The study was designed specifically to address testing and not vaccination uptake or hesitancy. We have added in the introduction that the vaccine became available in the middle of our formative research and therefore we decided we needed to add those questions to our focus group guide.*

**REVIEWER**. Methods: The authors might consider briefly explaining the integrated behavioral model. Many more details are needed on the specific interview questions and topics covered in the focus groups.

***RESPONSE.*** *This is addressed in the updated Methods. Topics covered are in the focus group interview guide which is available as a supplement. We do outline the key topics in the methods section.*
 **REVIEWER.** More information on the CBO-academic partnerships would be of interest and relevance to other researchers

***RESPONSE.*** *We have expounded on this partnership in the Methods section under the Study Setting subheading.*

**REVIEWER** Results:- The authors should consider first describing the demographic composition of their focus group participants

***RESPONSE.*** *As previously discussed, no other demographic information beyond race/ethnicity were collected under suggestion by a CBO partner because of concern that too many personal questions may be considered intrusive by the study population. This is a limitation of the study.*

**REVIEWER.**
- A lot of the wording in this section is awkward/clunky, i.e. "reasons for getting or not getting tested"
- "testing data storage and access" reads in a confusing way, the authors might instead consider "storage of and access to test results" A lot of the sentences do not use parallel structure, which impedes readability, i.e. "Residents suggested disseminating information through flyers, social media posts and emails, and that the information be translated into multiple languages." and "They also described setting up information tables in the developments, sending information to residents’ homes, and some suggested knocking on doors." ***RESPONSE.*** *We thank the reviewer for this comment and have revised to improve clarity.*

**REVIEWER** The authors should avoid interpreting in Results rather than Discussion, ie "This comment demonstrates the potential negative impact of misinformation often spread by word of mouth."
***RESPONSE.*** *We have removed this commentary from the Results section.*

**REVIEWER**- It is unclear how the authors classified and distinguished fears of testing from misinformation from mistrust, since there seemed to be a lot of overlap across the three

***RESPONSE.*** *These themes are not mutually exclusive, and often times in analysis a quote could be considered part of multiple domains. As reflected in our paper, fear of testing can encompass both practical issues* *(fear of a positive result, fear of pain as a result of the nasal swab), and outcomes that may be a result of mistrust or even misinformation. Misinformation as a separate domain can refer to untrue beliefs that residents hold about COVID-19 and testing, that don’t necessarily precipitate fear, i.e., that COVID-19 is “fake.”*

**REVIEWER** - Why were the responses from the predominantly Black group compared with the one Spanish speaking group, rather than with participants of other races in any group?
***RESPONSE.*** *Across all English-speaking focus groups, each group was predominantly or entirely comprised of non-Hispanic Black participants, so comparison of responses across other races is not feasible given the limited data.*

**REVIEWER** Discussion:
- "general lack of knowledge about testing recommendations, a similar lack of knowledge about risk factors for infection," - this was not presented at all in the Results
- The authors should discuss similar interventions that have been effective

**RESPONSE:** We have revised this sentence.

**REVIEWER** - It would be helpful for the authors to suggest more concrete solutions to mitigating mistrust and increasing access (ie transportation, ensuring that tests are free, etc.)
***RESPONSE:*** *Our solutions were those pulled directly from FG participants, which are reported in the Results section.*

**REVIEWER** - The authors need a limitations paragraph, especially detailing the potential biases (ie voluntary response, potential underrepresentation/lack of representation of certain perspectives or subgroups), shortcomings of methods, limited generalizability to other populations, etc.

***RESPONSE.*** *This has been added to the discussion section.*

Sincerely,

Chigozirim Izeogu, MD MPH
