# Supplementary material for: Attitudes, perceptions, and preferences towards SARS CoV-2 testing and vaccination among African American and Hispanic public housing residents, New York City: 2020–2021
Source: PLoS One. 2023 Jan 19;18(1):e0280460. doi: 10.1371/journal.pone.0280460 (PMC9851504; doi:10.1371/journal.pone.0280460)
Supplement: Attachment — Submitted filename: RADx FG PLoS One_SecondRevision_Response to Reviewers.docx [file pone.0280460.s003.docx]

Dear Dr. Liu,

Thank you for reviewing our manuscript and for the opportunity to revise it to meet PLoS One’s publication standard. Please find listed below the comments submitted by the reviewers and our response to each comment in bolded italicized font. Please let us know if we can clarify anything further.

**Reviewer #1**: Thank you for making changes based on Reviewer recommendations. I believe the manuscript has been improved by your changes. I do have some additional relatively minor changes.

**REVIEWER**. Introduction. Besides mentioning that there are 'social and structural determinants of health' impacting COVID-19 experiences between populations, is there any literature that you can mention in the Introduction on the beliefs or experiences related to testing and vaccination willingness/hesitancy? This may not be in African American and Hispanic groups, but I do believe the Introduction should acknowledge some of the research in this space, which if there is very little in these groups, leads to the gap this study is aiming to address.
***RESPONSE.*** *We have added additional literature on SARS-CoV-2 testing hesitancy among Black adults in the United States to the Introduction, specifically lines 76-80.*

**REVIEWER**. Introduction. Line 56 - this should be 'populations'.
***RESPONSE.*** *We thank the reviewer for bringing this error to our attention and have edited our manuscript accordingly.*

**REVIEWER**. Methods. Line 102 - how were these neighborhoods and areas identified as having large infection rates?
***RESPONSE.*** *We have added a clarifying sentence to the Methods section to delineate how neighborhoods were selected for our study (lines 111-114).*

**REVIEWER**. Methods. Could you provide information on how the CSC fit in with the study – was it formed to conduct the study or was the study an offshoot of this wider approach?
***RESPONSE.*** *We have added additional details regarding the formation of the community steering committee, as well as a new citation directing readers to a recently published paper on the formation and function of the CSC (lines 133-135).*

**REVIEWER**. Methods. Line 126 - correct extra I in COVIID.
***RESPONSE.*** *We thank the reviewer for bringing this error to our attention and have edited our manuscript accordingly.*

**REVIEWER**. Discussion. Line 296 - remove the 1 from this sentence - seems to be a typo.
***RESPONSE.*** *We thank the reviewer for bringing this error to our attention and have edited our manuscript accordingly.*

**REVIEWER**. Discussion. The Discussion ends with a Limitations section. It would be a shame to end with this when the study has collected important data. I would recommend a short Conclusion summarising what was found and future research or public health initiatives that are suggested.
***RESPONSE.*** *We have added an additional paragraph to the end of our Discussion section (lines 405-418).*

**Reviewer #3**: I appreciate the attention of the authors to addressing all my comments, and I feel that the manuscript has significantly improved. Just one minor thing remaining:

**REVIEWER**. Introduction. The authors state that they added in the Introduction that the vaccine became available in the middle of their research and therefore they added those questions to the focus group guide. However, I only see that mentioned in the Methods. It is important to add this to the Introduction. Otherwise I am happy with the revisions made and recommend the manuscript for publication. No need to come back to me as long as the above addition to the Intro is made.
***RESPONSE.*** *We have added this to our revised manuscript (lines 105-108).*
